# A comprehensive cancer analysis investigating the oncogenic role of zinc finger protein 36 (*ZFP36*) in human tumors

**DOI:** 10.1038/s41598-026-42715-5

**Published:** 2026-03-08

**Authors:** Shuyuan Xie, Huidan Wu, Xuanwen Li, Bingye Wang, Yuxuan Liu, Congying Li, Qing Huang, Yang Yang, Shinong Gu

**Affiliations:** 1 College of Environment and Public Health, Xiamen Huaxia University, Xiamen, 361000 Fujian P.R. China; 2 Graduate School of Health Science, Department of Clinical Nutrition, Tianjin Beichen Traditional Chinese Medicine Hospital, Tian Jin, China

**Keywords:** *ZFP36*, Pan-cancer, Survival, Prognosis, Bioinformatics, Immunological, Tumour biomarkers, Tumour immunology

## Abstract

The RNA-binding protein *ZFP36* is involved in tumorigenesis. To systematically elucidate its pan-cancer role, we conducted an integrated analysis of *ZFP36* across malignancies, combining bioinformatic exploration with experimental validation. Utilizing datasets from TCGA, GEO, GTEx, HPA, CPTAC, GEPIA2, TIMER2, cBioPortal, and STRING, we employed bioinformatics methods to investigate the potential carcinogenic properties of *ZFP36*. This included examining correlations between *ZFP36* and gene expression, prognosis, gene mutation, immunohistochemistry staining, immune cell infiltration, and constructing an interaction network of 50 *ZFP36*-binding proteins. Additionally, we performed enrichment analysis of *ZFP36*-related partners. Furthermore, to validate key bioinformatic predictions, quantitative real-time PCR (qRT-PCR) was performed on paired cancer/normal cell lines (LIHC, LUAD, BRCA). *ZFP36* expression was found to be dysregulated in a cancer type-dependent manner, with significant upregulation observed in most tumor types analyzed, including BLCA, BRCA, LIHC, and LUAD. Furthermore, *ZFP36* demonstrated early diagnostic value across 33 types of tumors and showed varying associations with prognosis depending on the tumor type. *ZFP36* was also significantly associated with most immune-infiltrating cells in pan-cancer analyses. High *ZFP36* expression was linked to pathways related to tumor progression. Critically, these bioinformatic predictions were experimentally validated, as qRT-PCR confirmed the significant upregulation of *ZFP36* and its functional network genes (*SOCS3*, *JUN*, *SLC7A11*, *CSRNP1*) in LIHC, LUAD, and BRCA cell lines. This study provides a comprehensive pan-cancer analysis of *ZFP36*, integrating bioinformatics with experimental validation. We demonstrated its dysregulation across cancers in a type-dependent manner, correlations with immune infiltration and tumor-associated pathways. Critically, qRT-PCR experiments confirmed the significant co-upregulation of *ZFP36* and its functional network genes (*SOCS3*, *JUN*, *SLC7A11*, *CSRNP1*) in LIHC, LUAD, and BRCA cell lines. These findings establish *ZFP36* as a promising diagnostic and prognostic biomarker and suggest its role as a pivotal post-transcriptional regulator in tumorigenesis, supporting its potential for clinical application in cancer assessment.

## Introduction

*Zinc finger protein 36 (ZFP36)* family proteins are crucial RNA-binding proteins involved in the metabolic pathway of messenger RNA (mRNA)^1^. Commonly referred to by their abbreviation, *ZFP36*, these proteins are characterized by a structure that includes multiple cysteines and/or histidine’s, stabilized by zinc ions^2^. The *ZFP36* protein family is distinguished by two tandemly repeating CCCH-type zinc finger motifs^3^, which bind to adenylyl-uridine-rich elements in the 3’-untranslated region (3’ UTR) of mRNAs, leading to the decay of the target mRNA. This binding process attenuates specific mRNAs, contributing to the regulation of their stability and degradation.

Extensive clinical research has elucidated both the unique and overlapping functions of *ZFP36* family members, particularly in immune cells. These proteins play critical and redundant roles in T-cell quiescence and the post-transcriptional regulation of inflammatory genes through RNA binding^4,5^. The *ZFP36* gene family encodes RNA-binding proteins that promote the degradation of transcripts containing AU-rich elements^6^. In tumor cells, conserved co-regulated genomic expression patterns have been associated with *ZFP36* levels. Notably, expression levels of this gene network are significantly downregulated in cancers of the breast^7^, liver^8^, lung^9^, kidney^10^, and thyroid compared to normal tissues. Despite a wealth of clinical data^11^, no pan-carcinogenic link has been established between *ZFP36* and various tumor types.

Recent studies employing integrated bioinformatic and experimental approaches have begun to elucidate the roles of specific zinc finger proteins in tumor immunity and progression. For instance, *Zinc Finger Protein 695* (*ZNF695*) was identified as a potential prognostic biomarker whose expression correlates significantly with immune infiltration and poor survival in cervical cancer, highlighting the clinical relevance of this protein family in the tumor microenvironment^12^. Separately, the expression of zinc finger E-box binding homeobox factors *ZEB1* and *ZEB2*, key transcriptional regulators of epithelial-mesenchymal transition (EMT), has been shown to escalate with increasing histopathological grade in oral squamous cell carcinoma, directly linking them to cancer aggressiveness^13^. These findings collectively highlight the necessity for systematic pan-cancer analyses to elucidate the prognostic and immunological roles of understudied regulatory factors.

However, despite its established role as a crucial RNA-binding protein that post-transcriptionally regulates mRNAs involved in inflammation and cell fate, a comprehensive understanding of *ZFP36*’s pan-cancer profile, its association with immune infiltration across diverse tumors, and its potential as a unified prognostic biomarker remains lacking. Motivated by the methodological precedent and biological context set by studies on related factors, this study aims to systematically bridge this gap.

Our study leverages data from The Cancer Genome Atlas (TCGA) project and the Gene Expression Omnibus (GEO) database to present the first pan-carcinogenic characterization of *ZFP36*. To explore the potential molecular mechanisms and clinical significance of *ZFP36* in the etiology or prognosis of diverse malignancies, we incorporated an array of variables, including gene expression, survival status, gene modifications, immunohistochemistry, immune infiltration, and associated cellular pathways.

## Materials and methods

### Gene expression analysis

*ZFP36* expression was analyzed using the “Gene DE” module of the TIMER2 (Tumor Immune Estimation Resource, version 2) platform (http://timer.cistrome.org/). Our analysis revealed significant differences in *ZFP36* expression between tumor tissues and their adjacent normal counterparts across various cancer types and specific tumor subtypes within the TCGA dataset. Following this, we expanded our analysis to include normal tissues that are either non-standard or particularly limited in availability, such as those from TCGA-GBM (glioblastoma multiforme) and TCGA-SKCM (cutaneous melanoma). For this purpose, we utilized the “Expression” module of the gepia2 (Gene Expression Profiling Interactive Analysis, version 2) web tool (http://gepia2.cancer-pku.cn/#analysis), which facilitated a comprehensive analysis of *ZFP36* expression in the TCGA dataset. Expression differences were visualized using box plots generated through the “Expression Analysis – Box Plot” module, comparing tumor tissues with corresponding normal tissues from the GTEx (Genotype-Tissue Expression) database. Statistical significance was determined using a *p*-value threshold of 0.01, with a log2FC (fold change) threshold set at 1, ensuring robust comparisons by matching TCGA normal data with GTEx data. Additionally, the “Pathology Stage Map” module of GEPIA2 was employed to generate violin plots, depicting *ZFP36* expression levels across different pathological stages (I-IV) in TCGA tumors.

### Survival prognosis analysis

We employed the “Survival Particles” module of GEPIA2 to generate significance plots for *ZFP36*, assessing its impact on overall survival (OS) and disease-free survival (DFS) across all TCGA tumor types. To further elucidate the prognostic value of *ZFP36*, where patients were stratified into high and low *ZFP36* expression groups based on the median expression value calculated separately for each cancer type (top 50% and bottom 50%). This standard, data-driven threshold was selected to ensure objective, reproducible, and balanced group assignment for survival comparison, in line with common practice in large-scale oncogenomic analyses. Survival analysis was conducted using the “Survival Analysis” module of GEPIA2, where survival curves were generated and compared using the log-rank test for hypothesis testing. Additionally, risk ratios and log-rank *p*-values were computed, incorporating the purity-adjusted Spearman rank correlation test along with other relevant statistical analyses to ensure the robustness of the findings.

### Genetic alteration analysis

To investigate the genetic alterations of *ZFP36*, we accessed the cBioPortal platform (https://www.cbioportal.org/) and selected the “TCGA Pan-Cancer Atlas Study” from the “Quick Choice” section. Within this dataset, we queried the gene alteration profile of *ZFP36* to obtain comprehensive information on its alteration landscape. Subsequently, we utilized the “Cancer Type Summary” module to analyze the alteration frequency, mutation types, and copy number alterations (CNA) across all TCGA tumors. The “Mutation” module allowed us to visualize *ZFP36* mutation sites within the context of the protein structure, both in schematic and three-dimensional (3D) formats. Additionally, the “Comparison” module was employed to assess the impact of *ZFP36* alterations on clinical outcomes, including overall survival, disease-specific survival, disease-free survival, and progression-free survival in TCGA cancer cohorts. Kaplan-Meier survival curves were generated, and statistical significance was evaluated using log-rank *p*-values.

### Immunohistochemistry (IHC) Staining

To evaluate *ZFP36* protein expression across different tissue types, we downloaded immunohistochemistry (IHC) images from the Human Protein Atlas (HPA) (http://www.proteinatlas.org/). The analysis included both normal tissues and six tumor types: lung adenocarcinoma (LUAD), breast invasive carcinoma (BRCA), ovarian serous cystadenocarcinoma (OV), liver hepatocellular carcinoma (LIHC), testicular germ cell tumors (TGCT), and thyroid carcinoma (THCA). It is important to note that HPA IHC data are semi-quantitative, based on staining intensity scores, and the sample size is typically small. Therefore, this analysis serves as a visual, qualitative reference for protein expression rather than a rigorous quantitative comparison.

Meanwhile, to validate *ZFP36* protein expression in alignment with our pan-cancer analysis, we selected six tumor types (LUAD, BRCA, OV, LIHC, TGCT, THCA) for IHC based on the following criteria: (i) confirmation of significant mRNA dysregulation in our prior analyses (LIHC, LUAD, BRCA), and (ii) extension to additional cancers (OV, TGCT, THCA) showing notable expression alterations in bioinformatic data to ensure broader representativeness across different organ systems.

### Immune infiltration analysis

We explored the relationship between *ZFP36* expression and immune infiltration across all TCGA cancers using the “immune gene” module of the TIMER2 web server. Our analysis focused on tumor-associated fibroblasts and T cells, given their critical roles in the tumor microenvironment. To estimate immune infiltration levels, we employed multiple computational algorithms, including TIMER, CIBERSORT, CIBERSORT-ABS, QUANTISEQ, XCELL, MCPCOUNTER, and EPIC. The use of multiple algorithms with diverse computational foundations was intended to provide a more comprehensive and robust assessment of immune infiltration, reducing reliance on any single estimation method. And the association between *ZFP36* expression and immune infiltration was quantified using the purity-adjusted Spearman rank correlation test to calculate *p*-values and partial correlation (cor) values. The results were visualized using heatmaps and scatter plots for clearer interpretation.

### *ZFP36*-related gene enrichment analysis

We conducted a *ZFP36*-related gene enrichment analysis using the STRING database (https://string-db.org). The search was performed with “Homo sapiens” as the species and “*ZFP36*” as the query protein. The analysis parameters were set as follows: a minimum required interaction score of “Low confidence (0.150),” the meaning of network edges as “evidence,” and a maximum of 50 interactors displayed in both the first and second interaction shells. A minimum interaction score of ‘Low confidence’ was selected to construct a comprehensive network of potential *ZFP36*-binding partners, facilitating an exploratory analysis aimed at hypothesis generation. The resulting network is inclusive and should be interpreted as a map of potential interactions for future investigation. Only experimentally validated interaction sources were included. This approach allowed us to identify and retrieve experimentally confirmed *ZFP36*-binding proteins.

Based on datasets of all TCGA^14^ cancers and normal tissues, we used the “Similar Gene Detection” module of GEPIA2 to retrieve the top 100 *ZFP36*-correlated targeting genes. We also carried out a pairwise gene Pearson correlation study of *ZFP36* and certain genes using the “correlation analysis” module of GEPIA2. For the dot plot, the log^2^ TPM was used. There were indications of the *p*-value and the correlation coefficient (R). Also, we utilized TIMER2^15^’s “Gene Corr” module to provide the heatmap data of the chosen genes, which includes the partial correlation (cor) and *p-* value in the purity-adjusted Spearman’s rank correlation test.

### Quantitative real-time PCR (qRT-PCR) analysis

The analysis was conducted across three cancer types (LIHC, LUAD, BRCA) using paired cancer/normal cell lines: HepG2 vs. THLE-2 for LIHC; A549 vs. BEAS-2B for LUAD; and MCF-7 vs. MCF-10 A for BRCA. All cell lines were purchased from Beyotime Biotechnology (Shanghai, China), with product numbers C6346 (HepG2), ST001 (THLE-2), C6053 (A549), C6106 (BEAS-2B), C6547 (MCF-7), and C6543 (MCF-10 A). Total RNA was extracted from cultured cells with Trizol reagent (Xinjing Bio, China). Cells in 6-well plates were lysed in 1 mL Trizol, mixed with 200 µL Buffer EX, and centrifuged at 12,000 × g for 15 min. The aqueous phase was transferred to isopropanol for RNA precipitation. The pellet was washed with 75% ethanol, air-dried, and dissolved in RNase-free water. RNA quality and concentration were assessed.

The cDNA was synthesized from 1 µg total RNA using the SureScript First-Strand cDNA Synthesis Kit (GeneCopoeia, USA) in a 20 µL reaction (Table 1). The reaction was incubated at 25 °C for 5 min, 42 °C for 15 min, and 85 °C for 5 min. And qPCR was performed using 2× SYBR Green Master Mix (Servicebio, China) on a SLAN-96 S system (Hongshi Medical, China). Primers for *ZFP36* and related genes (*SOCS3*, *JUN*, *SLC7A11*, *CSRNP1*) and the reference gene *GAPDH* (Table 2) were designed with Primer3Plus (https://www.primer3plus.com/) and validated with NCBI Primer-BLAST (http://www.ncbi.nlm.nih.gov/tools/primer-blast/). Then, the 20 µL qPCR reaction mix was prepared as in Table 2. Cycling conditions: 95 °C for 30 s; 40 cycles of 95 °C for 15 s and 60 °C for 30 s. Melt curve analysis (60 °C to 95 °C) confirmed specificity. All samples were run in triplicate.

**Table 1:** Reverse transcription reaction setup.

Component	Volume
SureScript RTase Mix (20×)	1.0 µL
SureScript RT Reaction Buffer (5×)	4.0 µL
Total RNA	1 µg
dd H_2_O (RNase/DNase free)	to 20µL

Noted: The reaction was performed to synthesize cDNA from 1 µg of total RNA. Incubation: 25 °C (5 min), 42 °C (15 min), 85 °C (5 min).

**Table 2:** qPCR primer sequences and reaction setup.

Target Gene	Forward Primer (5‘→3’)	Reverse Primer (5‘→3’)
*ZFP36*	CATGGATCTGCCTGGTGAAG	TCTGGCTGTCCTTGAGATGG
*SOCS3*	GCCTCAAGACCTTCAGCTCC	TGTCGCGGATCGTACTGGAT
*JUN*	AGTCCAGCAGATCGAGACC	CCTTCTGCACTGCTGAGGTT
*SLC7A11*	TCTCCAAAGGAGGTTACCTGC	AGACTCCCCTCAGTAAAGTGAC
*CSRNP1*	AACGGAAGACCTGGACAAGG	GGCTGTAACCGTCCTTGTTG
*GAPDH*	GGAGCGAGATCCCTCCAAAAT	GGCTGTTGTCATACTTCTCATGG
Component	Volume per 20 µL reaction	Final Concentration
2×SYBR Premix Ex Taq	10.0 µL	1$\times$
F Primer (5 pmol/ µL)	2.0 µL	0.5µM
R Primer (5 pmol/ µL)	2.0 µL	0.5µM
cDNA template	2.0 µL	-
dd H_2_O (RNase/DNase free)	4.0 µL	-

Noted: Primers were designed with Primer3Plus and validated by NCBI Primer-BLAST. The qPCR assay was conducted in triplicate using SYBR Green Master Mix (SLAN-96 S system). Thermal cycling: 95 °C for 30 s, followed by 40 cycles of 95 °C for 15 s and 60 °C for 30 s.

The relative gene expression (fold change) was calculated using the comparative ΔΔCT method^16^, with the formula: 2^−ΔΔCT^, where ΔCT = CT (target) – CT(GAPDH) and ΔΔCT = ΔCT (experimental) – ΔΔCT (control). Data from three independent experiments are presented as mean ± SD. Statistical analysis and graph generation were performed using GraphPad Prism software (10.1.2 version, USA), with one-way ANOVA used to compare differences between cancer cell lines (HepG2, A549, MCF-7) and their corresponding normal cell lines (THLE-2, BEAS-2B, MCF-10 A). A *p*-value < 0.05 was considered statistically significant.

## Results

### *ZFP36* expression is upregulated in multiple tumors

**Fig. 1:**
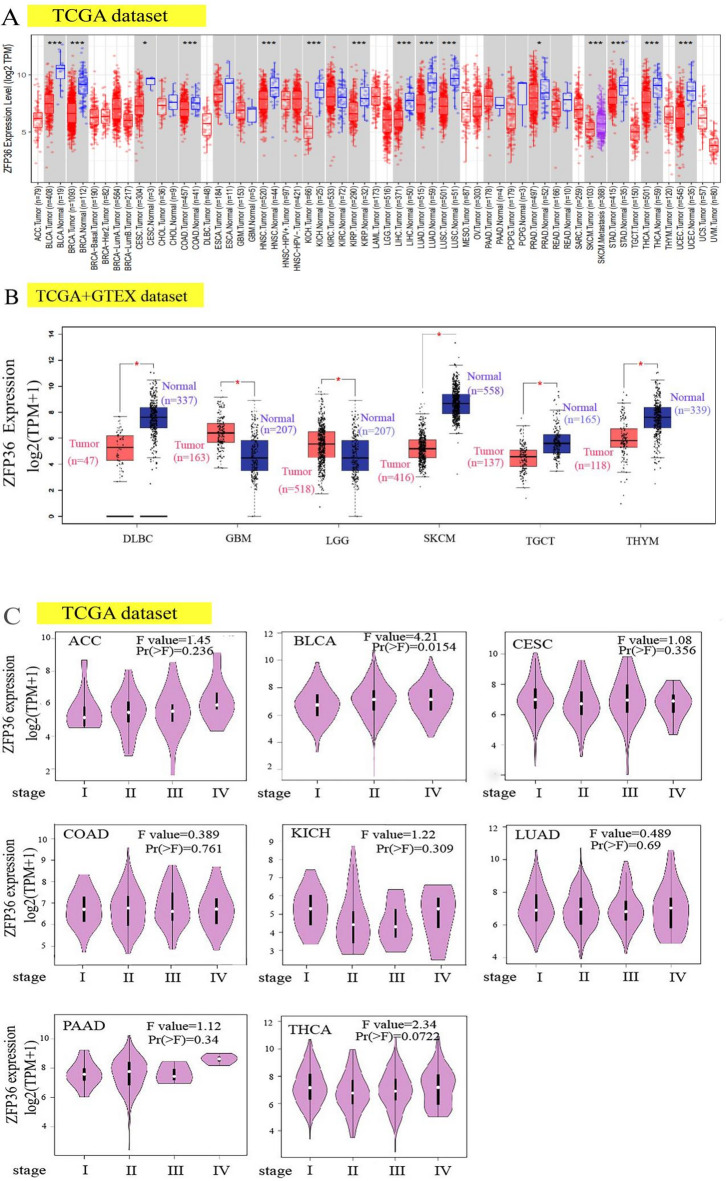
Expression level of* ZFP36* gene in different tumors and pathological stages. (**a**) Expression status of *ZFP36* gene in different cancers or specific cancer subtypes was analyzed by TIMER2. * *p* < 0.05; ** *p* < 0.01; ***p < 0.001. (**b**) For the TCGA program, DLBC (tumor *N* = 47; normal *N* = 337), GBM (tumor *N* = 163; normal *N* = 207), LGG (tumor *N* = 518; normal *N* = 207), SKCM (tumor *N* = 461; normal *N* = 558), TGCT (tumor *N* = 137; normal *N* = 165), and THYM (tumor *N* = 118; normal *N* = 165), the expression status of ZFP36 gene was determined. *N* = 137; normal *N* = 165), TGCT (tumor *N* = 137; normal *N* = 165), and THYM (tumor *N* = 118; normal *N* = 339) types, and the corresponding normal tissues from the GTEx database are included as controls. Box plot data are provided. ** *p* < 0.01.(**v**) Expression levels of the *ZFP36* gene were analyzed by major pathological stages (stage I, II, III, and IV) of ACC, BLCA, CESC, COAD, KICH, LUAD, PAAD, and THCA based on the TCGA data. log2 (TPM + 1) was applied to the logarithmic scale.

To assess *ZFP36* mRNA expression across various cancers, we analyzed combined tumor and normal samples from the TCGA dataset. As shown in Fig. 1A, *ZFP36* expression was elevated in several tumor types, including BLCA(*p* < 0.001), BRCA(*p* < 0.001), CESC(*p* < 0.05), COAD(*p* < 0.001), GBM, HNSC(*p* < 0.001), KICH(*p* < 0.001), KIRC, KIRP(*p* < 0.001), LIHC(*p* < 0.001), LUAD(*p* < 0.001), LUSC(*p* < 0.001), PAAD, PCPG, PRAD(*p* < 0.05), READ, STAD(*p* < 0.001), THCA(*p* < 0.001), and UCEC(*p* < 0.001). Notably, *ZFP36* expression was significantly higher in PAAD compared to normal tissues. To further investigate these findings, we used normal tissues from the GTEx dataset as controls to examine *ZFP36* expression differences between normal and tumor tissues in greater detail. As depicted in Fig. 1B, *ZFP36* expression was significantly lower in DLBC, SKCM, TGCT, and THYM compared to normal tissues, whereas it was markedly higher in GBM and LGG. As depicted in Fig. 1C, *ZFP36* expression was found to be associated with pathological stages in several cancers, including ACC, BLCA, CESC, COAD, KICH, LUAD, PAAD, and THCA. Specifically, we examined the correlation between *ZFP36* expression and the pathological stages of ACC and BLCA using the “Pathological Staging Map” module of GEPIA2 and visualized these correlations through violin plots. (Fig. 1D, p-value < 0.05 only for BLCA, *p*-value < 0.1 for THCA)

### *ZFP36* expression is related to the prognosis of various tumors

**Fig. 2:**
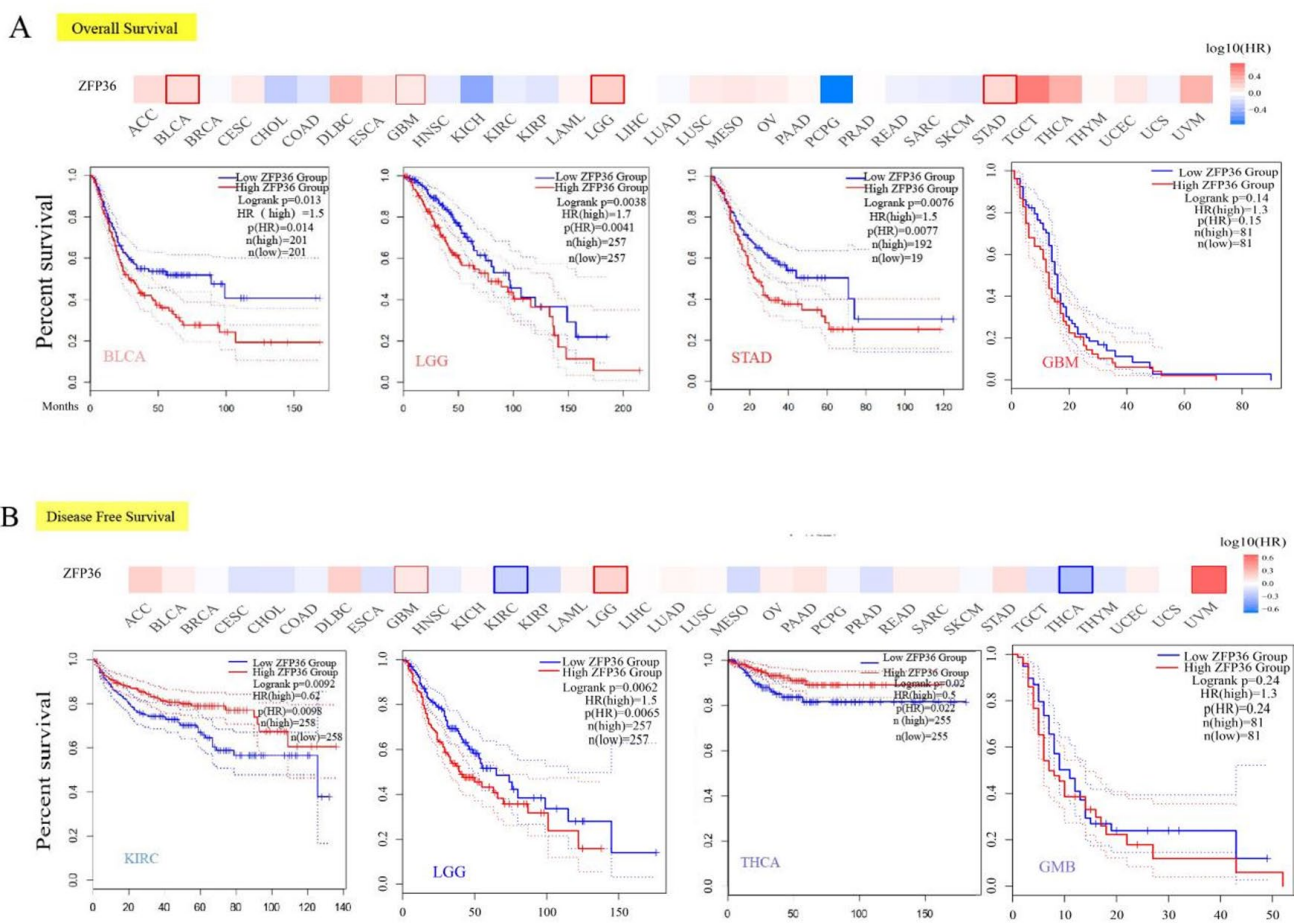
Correlation between* ZFP36* gene expression and survival prognosis of cancers in TCGA. We used the GEPIA2 tool to analyze overall survival (**a**) and disease-free survival (**b**) of different tumors in TCGA by *ZFP36* gene expression. Survival plots and Kaplan-Meier curves are given for positive results. Red labels indicate high *ZFP36* expression groups and blue labels indicate low *ZFP36* expression groups.

To investigate the relationship between *ZFP36* expression and patient prognosis across various cancers, we stratified tumor cases into two groups: high *ZFP36* expression and low *ZFP36* expression. As shown in Fig. 2A, our analysis of the TCGA dataset revealed that high *ZFP36* expression was significantly correlated with poorer OS in KIRC (*p* = 0.0092), LGG (*p* = 0.0038), and STAD (*p* = 0.0076). Similarly, DFS analysis, presented in Fig. 2B, indicated that elevated *ZFP36* expression was associated with a poor prognosis in LGG (*p* = 0.0038).

### The characteristics of *ZFP36* mutations in the TCGA pan-cancer cohort

**Fig. 3:**
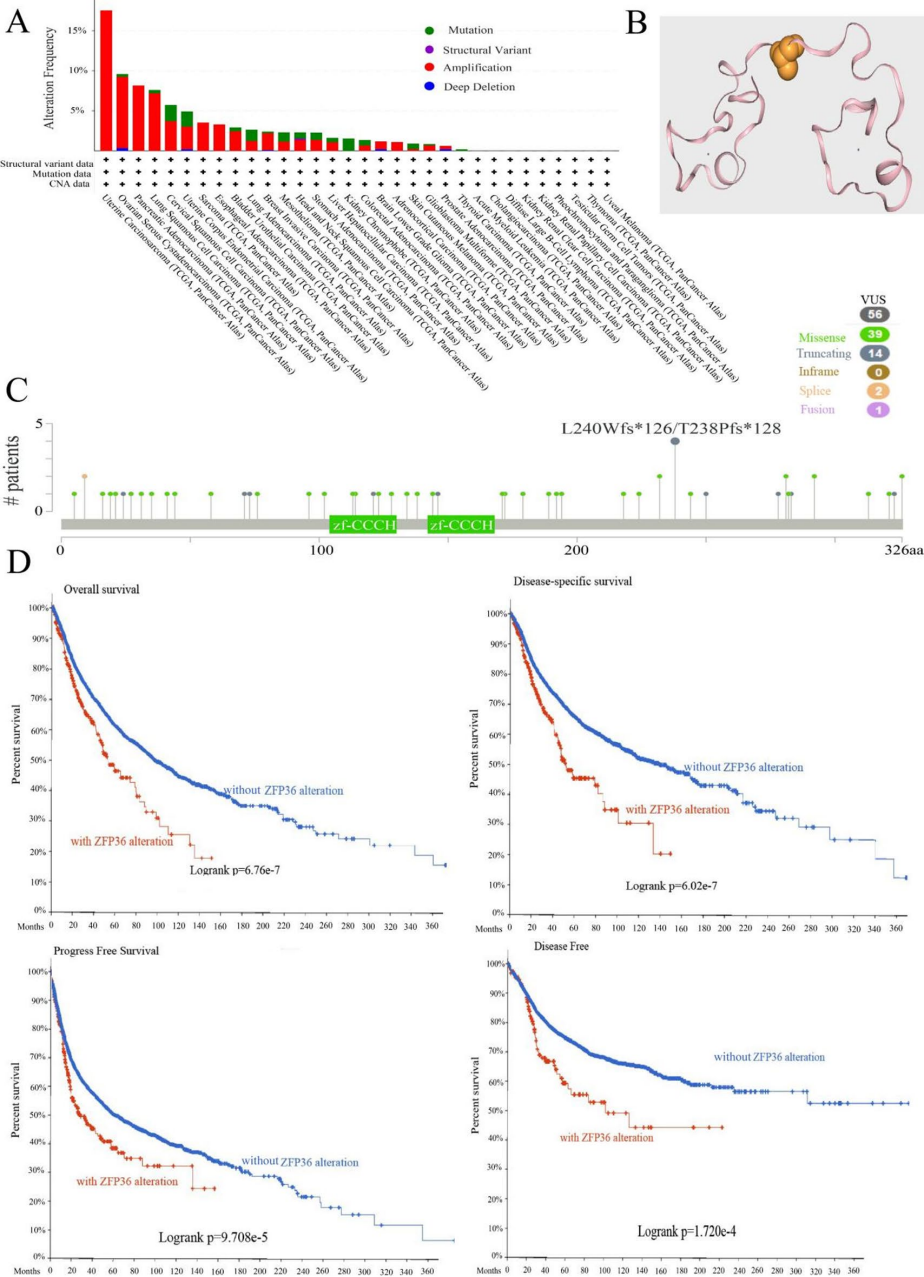
Mutation feature of* ZFP36* in different tumors of TCGA. We analyzed the *ZFP36* mutation profile of TCGA tumors using the cBioPortal tool. Alteration frequencies for mutation type (**a**) and mutation site (**b**) are shown. We show the mutation site with the highest alteration frequency (L240Wfs*126/T238Pfs*128) in the 3D structure of *ZFP36* (**c**). We also analyzed the potential correlation between mutation status and (d) overall, disease-specific, disease-free and progression-free survival using the cBioPortal tool.

We analyzed the genetic status of *ZFP36* across various tumor samples from the TCGA cohort. As illustrated in Fig. 3A, patients with UCEC tumors exhibited the highest “Alteration Frequency” (> 15%), with the alterations in UCEC, ESCA, PAAD, and SARC primarily consisting of “Amplification.” Notably, the “Alteration Frequency” in KICH patients was exclusively due to “Mutation.” Additionally, OV cases showed an “Alteration Frequency” between 5% and 10%, predominantly composed of “Amplification,” with minor contributions from “Mutation” and “Deep Deletion” (Fig. 3A). Cases of LUSC and CESC with genetic alterations predominantly displayed *ZFP36* amplification (Fig. 3A). The types, loci, and frequencies of *ZFP36* mutations are detailed in Fig. 3C, where missense mutations were found to be more prevalent compared to other genetic alterations. The three-dimensional structure of *ZFP36* is presented in Fig. 3B. Survival analysis, as depicted in Fig. 3D, revealed that UCEC patients with *ZFP36* gene alterations had better PFS (*p* = 9.708e-5), DSS (*p* = 6.02e-7), and OS (*p* = 6.76e-7), while DFS (*P* = 1.720e-4) was associated with a worse prognosis. This figure effectively demonstrates the relationship between *ZFP36* genetic alterations and clinical survival outcomes across different cancer types.

### Protein expression analysis of *ZFP36* via Immunohistochemistry

**Fig. 4:**
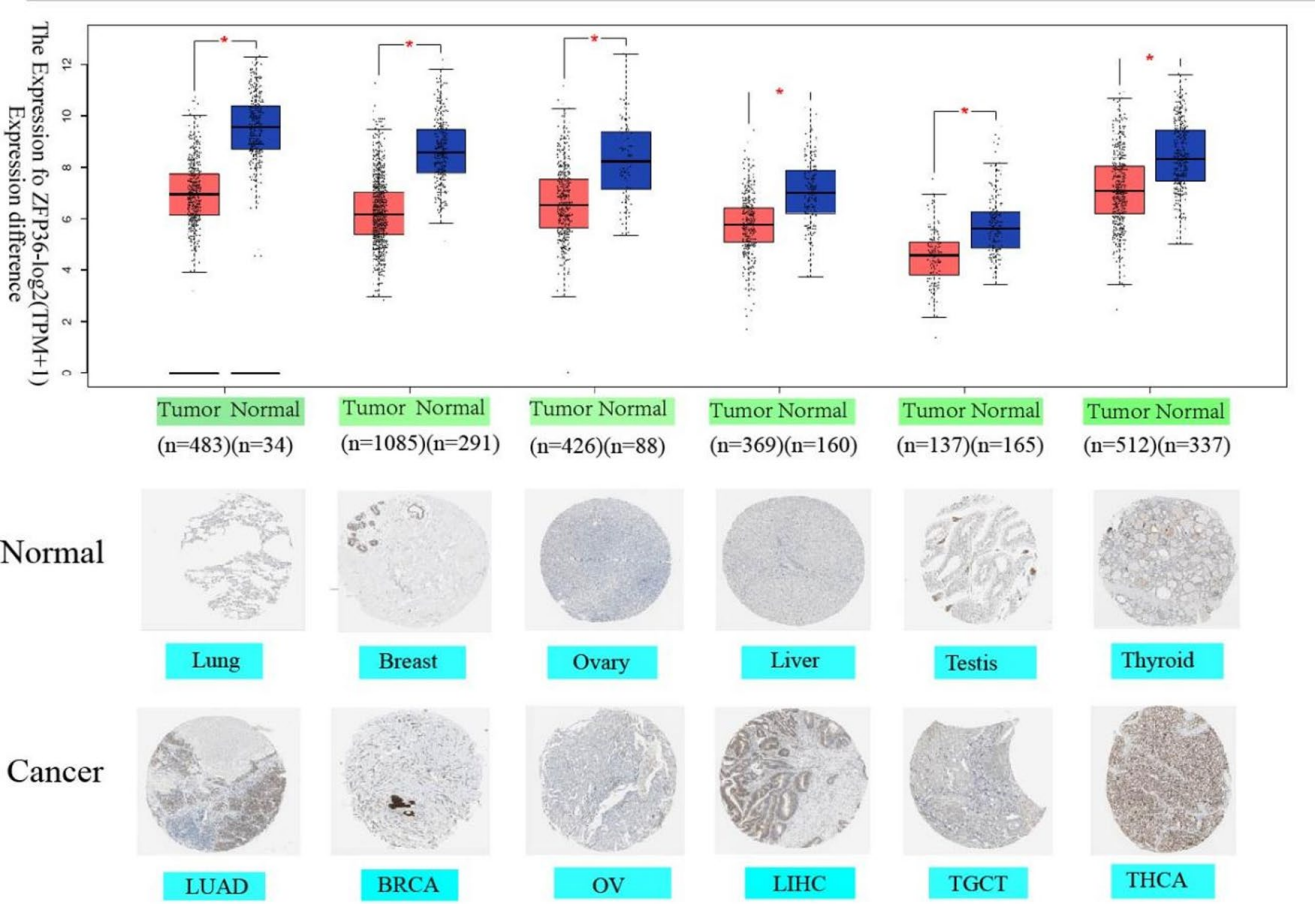
Gene expression and immunohistochemistry of* ZFP36* in tumors and normal tissues. For the LUAD, BRCA, OV, LIHC, TGCT, and THCA types in the TCGA project, the corresponding normal tissues from the GTEx database are included as controls. Box plot data are provided. * *p* < 0.05. We also obtained immunohistochemical results of *ZFP36* in tumor and normal tissues using the HPA database. Protein expression of GPC2 in immunohistochemical images of normal (top) and tumor (bottom) groups. (**a**) lung, (**b**) breast, (**c**) ovary, (**d**) liver, (**e**) testis, (**f**) thyroid.

The immunohistochemical analysis revealed distinct expression patterns of at the protein level. As shown in Fig. 4, the IHC staining intensity indicated variable *ZFP36* protein levels, with semi-quantitative scores suggesting a trend towards higher expression in some normal tissues compared to their tumor counterparts in LUAD, BRCA, OV, LIHC, TGCT, and THCA. This observation, while providing a protein-level snapshot, should be interpreted with caution due to the semi-quantitative nature and limited sample size of HPA data.

### Immunoinfiltration analysis of *ZFP36* and Its prognostic implications

Previous studies have indicated that cancer-associated fibroblasts in the tumor microenvironment play a crucial role in modulating immune cell infiltration. To explore the relationship between cancer-associated fibroblast infiltration and *ZFP36* expression across various malignancies, we utilized algorithms such as EPIC, MCPCOUNTER, and XCELL.

**Fig. 5:**
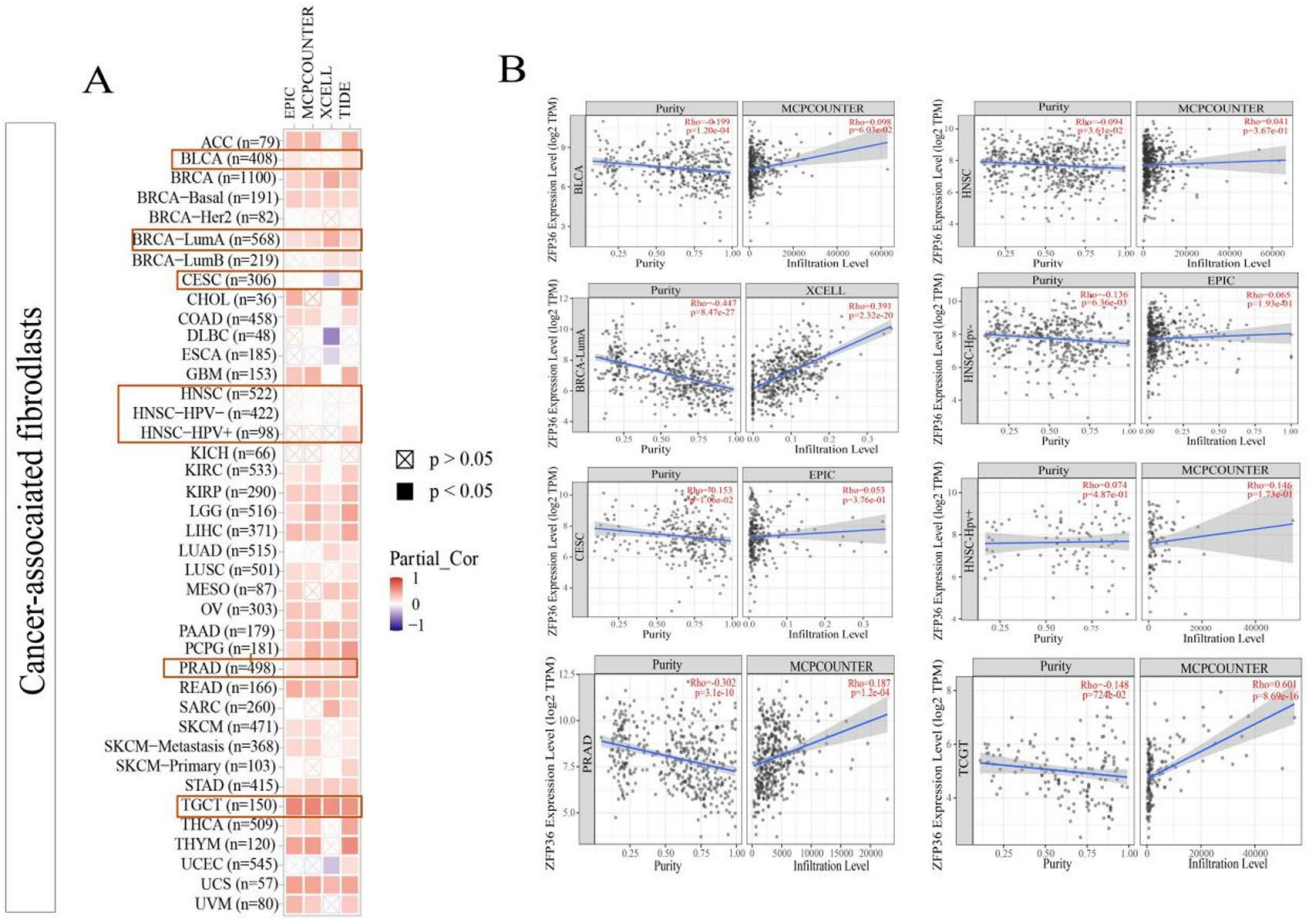
Correlation analysis between* ZFP36* expression and immune infiltration of cancer-associated fibroblasts. Different algorithms (EPIC, MCPCOUNTER, and XCELL) were used to explore the potential correlation between the level of *ZFP36* gene expression and the level of cancer-associated fibroblast infiltration in all types of cancers in TCGA. (**a**) Heatmap representing the correlation between *ZFP36* and tumor-associated fibroblast infiltration. (**b**) The graph is a scatter plot representing the correlation between *ZFP36* and tumor-associated fibroblast infiltration.

As depicted in Fig. 5, the scatter plots generated by these algorithms reveal correlations. Notably, according to the MCPCOUNTER algorithm, a significant positive correlation between *ZFP36* expression and cancer-associated fibroblast infiltration was observed in PRAD (Rho = 0.187, *p* = 0.012) and TGCT (Rho = 0.601, *p* = 8.69e-16). In contrast, no statistically significant correlations were found in BLCA (Rho = -0.098, *p* = 0.0603), HNSC (Rho = 0.041, *p* = 0.367), HNSC-HPV+ (Rho = 0.146, *p* = 0.100), and CESC (Rho = 0.053, *p* = 0.376). Furthermore, the XCELL algorithm indicated a positive correlation between *ZFP36* expression in cancer-associated fibroblasts and BRCA-LumA (Rho = 0.391, *p* = 2.32e-20). The EPIC algorithm also identified a positive correlation for *ZFP36* expression in cancer-associated fibroblasts from HNSC-HPV- (Rho = 0.065, *p* = 0.193), although this did not reach statistical significance. These findings suggest that ZFP36 may play a significant role in the tumor microenvironment in a context-dependent manner, with particularly strong associations observed in TGCT, PRAD, and BRCA-LumA.

While the estimated correlation coefficients varied across algorithms, which is a recognized aspect of in silico deconvolution, the consistent identification of significant associations (e.g., in TGCT and BRCA-LumA) by independent methods supports a robust link between *ZFP36* expression and immune contexture.

### Enrichment Analysis Implicates “Hemopoiesis” in *ZFP36*-mediated tumor pathogenesis

**Fig. 6:**
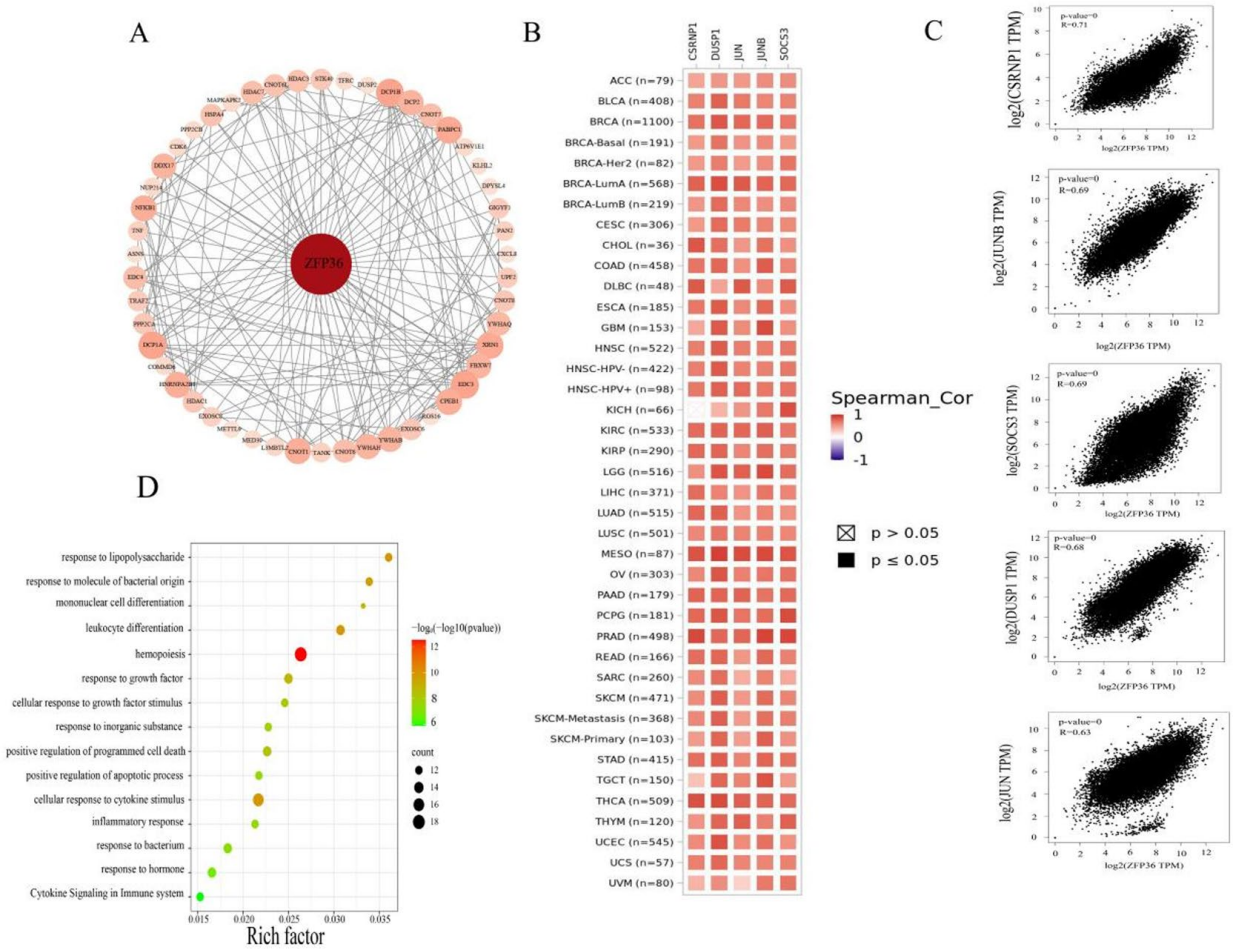
*ZFP36*-related gene enrichment analysis. (**a**) We first used the STRING tool to obtain available experimentally determined *ZFP36* binding proteins. (**b**) Corresponding heatmap data in detailed cancer types are shown. (**c**) Using the GEPIA2 method, we also obtained the top 100*ZFP36*-associated genes in the TCGA project and analyzed the expression correlation between *ZFP36* and selected target genes, including *CSRNP1*, *JUNB*, *SOCS3*, *DUSP1* and *JUN*. (**d**) KEGG pathway analysis based on *ZFP36* binding and interacting genes.

To elucidate the molecular mechanisms by which *ZFP36* influences carcinogenesis, we conducted pathway enrichment studies focused on *ZFP36*-binding proteins and related genes. A total of 50 experimentally validated *ZFP36*-binding proteins were identified, and their interaction network is presented in Fig. 6A. Using the GEPIA2 program, we analyzed the top 100 genes associated with *ZFP36* expression across TCGA tumor datasets. Notably, a significant correlation was observed between *ZFP36* expression and *CSRNP1* (Fig. 6C). In various cancer types, strong positive correlations were found between *ZFP36* and several genes: *CSRNP1* (*R* = 0.71), *JUNB* (*R* = 0.69), *SOCS3* (*R* = 0.69), *DUSP1* (*R* = 0.68), and *JUN* (*R* = 0.63), all with *p*-values < 0.01. The associated heatmap data further corroborated these relationships (Fig. 6B).

Additionally, EGG enrichment analysis integrating two datasets, indicated that pathways related to “Hematopoietic cell lineage” (hemopoiesis) may be associated with *ZFP36*-related tumor pathogenesis, as illustrated in Fig. 6D. While the direct link between “hemopoiesis” and solid tumors is not immediately apparent, this enrichment could reflect the well-established role of *ZFP36* in regulating immune cell development and function. Given that tumor-infiltrating immune cells are of hematopoietic origin, this pathway association may indirectly point to *ZFP36*’s role in modulating the tumor immune microenvironment.

### Experimental validation of *ZFP36* and its functional network gene expression in cancer cell lines

The mRNA expression levels of *ZFP36*, *SOCS3*, *JUN*, *SLC7A11*, and *CSRNP1* were quantitatively assessed by qRT-PCR in three cancer types: hepatocellular carcinoma (LIHC: HepG2 vs. THLE-2), lung adenocarcinoma (LUAD: A549 vs. BEAS-2B), and breast cancer (BRCA: MCF-7 vs. MCF-10 A). Relative expression was calculated using the 2^−ΔΔCt^ method with normalization to a housekeeping gene.

**Fig. 7:**
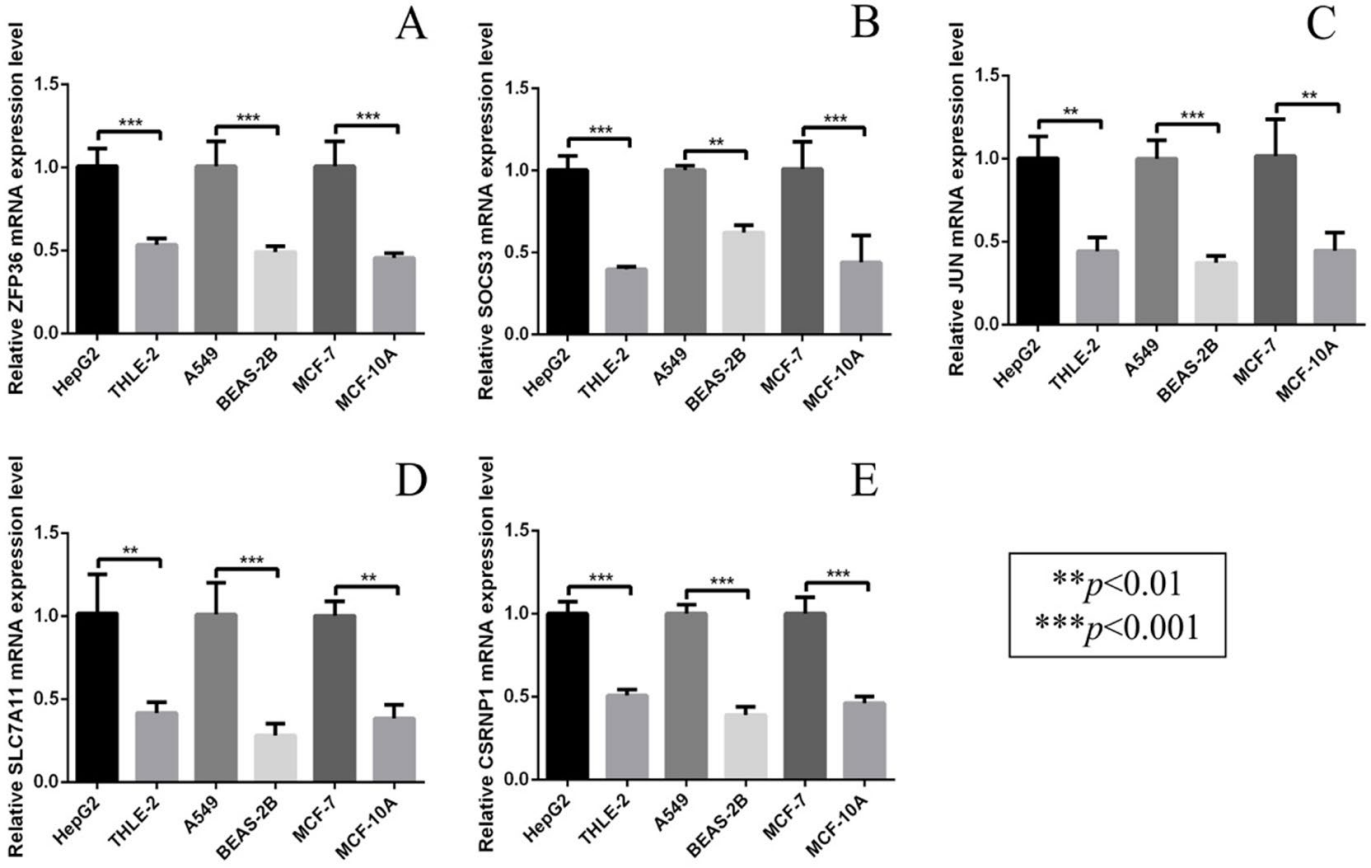
qRT-PCR validation of *ZFP36*-related gene expression in multiple cancer cell lines. (**a**) Relative mRNA expression levels of *ZFP36* in LIHC (HepG2), LUAD (A549), and BRCA (MCF-7) cell lines compared with their corresponding normal epithelial cells (THLE-2, BEAS-2B, and MCF-10 A), as determined by qRT-PCR. (**b**) Relative mRNA expression levels of *JSOCS3* in LUAD, LIHC, and BRCA cell lines compared with matched normal epithelial cells. (**c**) Relative mRNA expression levels of *JUN* in LUAD, LIHC, and BRCA cell lines compared with matched normal epithelial cells. (**d**) Relative mRNA expression levels of *SLC7A11* in LUAD, LIHC, and BRCA cell lines compared with matched normal epithelial cells. (**e**) Relative mRNA expression levels of *CSRNP1* in LUAD, LIHC, and BRCA cell lines compared with matched normal epithelial cells. Statistical significance is indicated as **p < 0.01 and ***p < 0.001.

As shown in Fig. 7, all five target genes were consistently and significantly upregulated in the cancer cell lines compared to their corresponding normal epithelial controls. The expression of *ZFP36* was markedly elevated across all three cancer models (all **p < 0.001, Fig. 7A). Similarly, *SOCS3*, *JUN*,* SLC7A11*, and *CSRNP1* showed significant increases in expression in HepG2, A549, and MCF-7 cells, with statistical significance ranging from **p < 0.01 to **p < 0.001 (Fig. 7B-E). This coherent upregulation pattern suggests that these genes may play important and convergent roles in the pathogenesis of diverse cancers.

## Discussion

RNA-binding proteins are crucial regulators of T cell activation, proliferation, and cytokine production^17^. The *ZFP36* family encodes such proteins, facilitating the degradation of transcripts containing AU-rich elements^6^. Numerous studies have highlighted the distinct and overlapping functions of the *ZFP36* family in immune cells, revealing that *ZFP36* proteins play both redundant and critical roles in regulating T cell quiescence^17^. In clinical trials, *ZFP36* disruption led to a modest increase in antigen-independent activation and cytokine responses; however, it did not adversely affect CAR-T cell function in xenograft models using NSG mice^18^. These findings suggest that a singular disruption of *ZFP36* may be insufficient to enhance functionality, indicating a potential benefit from a multiplexed approach.

In this study, we conducted a pan-cancer analysis of *ZFP36*, revealing its frequent upregulation at the mRNA level in a variety of tumors, including LIHC, LUAD, and BRCA. Our experimental validation further confirmed this upregulation in corresponding cancer cell lines, substantiating the bioinformatic findings.

Survival analysis revealed that *ZFP36* expression did not significantly impact overall survival (OS) or disease-free survival (DFS) in GBM patients (*p* = 0.14 and *p* = 0.24, respectively). Conversely, in LGG patients, high ZFP36 expression correlated with poorer OS (*p* = 0.0038) and DFS (*p* = 0.0062), suggesting that *ZFP36* expression levels could serve as important prognostic indicators for this cancer type. Meanwhile, *ZFP36*’s association with poor prognosis in LGG aligns with previous findings that *ZFP36L2* is overexpressed in LGG and linked to adverse clinical outcomes^19^. Additionally, a study identified *ZFP36* among 14 genes associated with LGG progression and drug resistance pathways, underscoring its potential as a target for personalized immunotherapies aimed at extending patient survival^20^.

In GBM, despite the lack of a significant impact on OS or DFS based on *ZFP36* expression, the role of iron death resistance is crucial. Recent research has shown that DLEU1, an oncogenic lncRNA, binds to *ZFP36* and facilitates the degradation of ATF3 mRNA, thereby upregulating SLC7A11 and promoting resistance to iron death^21^. Interestingly, our own experimental data also demonstrated a significant upregulation of *SLC7A11* in LIHC, LUAD, and BRCA cell lines, suggesting that the *ZFP36/SLC7A11* axis might be a conserved mechanism across multiple tumor types beyond GBM.

The lack of a significant correlation between elevated *ZFP36* expression and patient survival in GBM, contrasting with its clear prognostic value in LGG, invites further mechanistic consideration. Several factors inherent to GBM biology may contribute to this discrepancy. First, the profound intratumoral heterogeneity and immunosuppressive microenvironment of GBM may render the impact of a single RNA-binding protein like *ZFP36* on clinical outcome less discernible, as it could be overshadowed by dominant drivers such as IDH mutation status or MGMT promoter methylation^22,23^. Second, functional redundancy within the *ZFP36* family (e.g., *ZFP36L1*,* ZFP36L2*) might allow for compensatory regulation in GBM, buffering the phenotypic consequences of *ZFP36* fluctuation^24^. Third, the role of *ZFP36* may be critically context dependent^25^. As noted earlier, in GBM, *ZFP36* can be recruited by oncogenic lncRNAs like DLEU1 to promote ferroptosis resistance via the ATF3/SLC7A11 axis^21^. This pro-survival function in a specific pathway might exist in parallel with other, potentially tumor-suppressive roles, leading to a net neutral effect on overall patient survival in multivariate analyses. This complexity underscores that the functional output of *ZFP36* is likely determined by the integrated molecular circuitry of the specific tumor type.

Further investigations have positioned *ZFP36* as a critical prognostic marker across various malignancies. For instance, *ZFP36* has been identified as a diagnostic marker in chronic kidney disease combined with non-alcoholic fatty liver disease^26^, while low levels of *ZFP36* have been associated with poor outcomes in gastric and prostate cancers^27^. The aberrant expression of *ZFP36* may significantly correlate with cancer progression and patient prognosis, suggesting its viability as a candidate biomarker.

Based on systematic bioinformatic analyses that predicted the pan-cancer expression pattern and clinical significance of *ZFP36*, we performed qRT-PCR experiments to validate these predictions and explore the functional context. Our experimental results demonstrated that *ZFP36* mRNA expression was significantly upregulated in LIHC, LUAD and BRCA cell lines, directly corroborating the core bioinformatic finding (Corresponding to Result 3.1). Notably, key genes functionally linked to *ZFP36* (*SOCS3*, *JUN*, *SLC7A11*, *CSRNP1*) also exhibited a consistent and coordinated upregulation across these cancer cell lines. This co-expression profile suggests that ZFP36 may collectively influence tumor progression by regulating related pathways such as immune response (e.g., *SOCS3*,* JUN*), ferroptosis resistance (*SLC7A11*), and cellular stress (*CSRNP1)*. These findings provide initial experimental support for the previously identified associations between *ZFP36* expression and tumor immune infiltration (Corresponding to Result 3.5), specific pathway enrichment (Corresponding to Result 3.6), and patient prognosis (Corresponding to Results 3.2, 3.3), placing these correlations within a more concrete and coherent molecular network framework.

While this study integrates bioinformatics with experimental validation, several limitations should be acknowledged. First, the immune infiltration analysis relied on computational algorithms; inherent discrepancies in the magnitude or significance of correlations are expected due to differences in their underlying gene signatures and models^29,30^. Therefore, these results are best interpreted as revealing consistent biological trends rather than precise quantitative metrics. Second, the protein-protein interaction network for *ZFP36* was constructed using an inclusive confidence threshold (0.150) from the STRING database. While this exploratory approach provided a broad landscape of potential interactors, it inherently includes associations that vary in confidence, serving primarily as a resource for generating mechanistic hypotheses. Third, and most importantly, our experimental validation has significant constraints. The qRT-PCR analysis was limited to mRNA levels and did not include protein-level validation (e.g., western blot or IHC). Furthermore, each cancer type was represented by only a single cell line (e.g., HepG2 for LIHC, A549 for LUAD, MCF-7 for BRCA), which may not fully capture the heterogeneity of primary tumors or their various subtypes. Future investigations employing a broader panel of cell lines, protein-level analyses, and functional assays (e.g., knockout/overexpression) are essential to confirm the precise role of *ZFP36* in tumorigenesis.

Collectively, to future directions and clinical implications, this study integrates computational prediction with experimental validation to reveal a consistent transcriptional upregulation pattern of *ZFP36* and its functional network genes in LIHC, LUAD, and BRCA. This finding suggests that *ZFP36* may act as a pivotal post-transcriptional regulatory node. By orchestrating the expression of this downstream gene set, *ZFP36* could drive analogous pro-tumorigenic phenotypes across molecularly distinct solid tumors. Such research could illuminate *ZFP36*’s role in tumorigenesis and progression, ultimately informing the development of more precise and personalized immunotherapeutic strategies^31^.

## Conclusions

In summary, this study provides a systematic pan-cancer characterization of *ZFP36* by integrating multidimensional bioinformatics with experimental validation. *ZFP36* expression was found to be frequently upregulated at the mRNA level in multiple tumors, including LIHC, LUAD, and BRCA. Its expression also correlated with immune infiltration and was linked to pathways such as “hemopoiesis cell lineage” through enrichment analysis of its interaction network. Critically, these computational predictions were substantiated by qRT-PCR experiments, which confirmed the significant co-upregulation of *ZFP36* and its key functional network genes (*SOCS3*,* JUN*, *SLC7A11*, *CSRNP1*) in LIHC, LUAD, and BRCA cell lines. Collectively, our findings position *ZFP36* not only as a potential prognostic biomarker with diagnostic value across multiple cancers but also as a pivotal post-transcriptional regulatory node that may orchestrate a conserved pro-tumorigenic gene program. These results indicate that ZFP36 may serve as a promising prognostic biomarker for clinical diagnosis and cancer assessment.

## Data Availability

All data analyzed in this study were obtained from the publicly accessible TCGA, CPTAC, and GEO databases, as referenced in the Methods. To ensure reproducibility, the custom analysis code and the key processed datasets generated are available at DOI 10.6084/m9.figshare.31333831.

## Abbreviations

ACC: Adrenocortical carcinoma
BLCA: Bladder urothelial carcinoma
BRCA: Breast invasive carcinoma
CESC: Cervical squamous cell carcinoma and endocervical adenocarcinoma
COAD: Colon adenocarcinoma
DLBC: Lymphoid neoplasm diffuse large B-cell lymphoma
ESCA: Esophageal carcinoma
GBM: Glioblastoma multiforme
HNSC: Head and neck squamous cell carcinoma
KICH: Kidney chromophobe
KIRP: Kidney renal papillary cell carcinoma
LGG: Brain lower grade glioma
LIHC: Liver hepatocellular carcinoma
LUAD: Lung adenocarcinoma
LUSC: Lung squamous cell carcinoma
OV: Ovarian serous cystadenocarcinoma
PAAD: Pancreatic adenocarcinoma
PCPG: Pheochromocytoma and paraganglioma
PEAD: Rectum adenocarcinoma
PRAD: Prostate adenocarcinoma
SARC: Sarcoma
SKCM: Skin cutaneous melanoma
STAD: Stomach adenocarcinoma
TGCT: Testicular germ cell tumors
THCA: Thyroid carcinoma
THYM: Thymoma
UCEC: Uterine corpus endometrial carcinoma
